# Mortality in Severe Human Immunodeficiency Virus-Tuberculosis Associates With Innate Immune Activation and Dysfunction of Monocytes

**DOI:** 10.1093/cid/cix254

**Published:** 2017-03-24

**Authors:** Saskia Janssen, Charlotte Schutz, Amy Ward, Elisa Nemes, Katalin A Wilkinson, James Scriven, Mischa A Huson, Nanne Aben, Gary Maartens, Rosie Burton, Robert J Wilkinson, Martin P Grobusch, Tom Van der Poll, Graeme Meintjes

**Affiliations:** 1 Clinical Infectious Diseases Research Initiative, Institute of Infectious Disease and Molecular Medicine, University of Cape Town, South Africa;; 2 Center of Tropical Medicine and Travel Medicine and; 3 Center for Experimental and Molecular Medicine, Department of Infectious Diseases, Division of Internal Medicine, Academic Medical Center, University of Amsterdam, Amsterdam, The Netherlands;; 4 Department of Medicine, Groote Schuur Hospital and; 5 South African Tuberculosis Vaccine Initiative, University of Cape Town, South Africa;; 6 Francis Crick Institute Mill Hill Laboratory, London, and; 7 Liverpool School of Tropical Medicine, United Kingdom;; 8 Computational Cancer Biology, Division of Molecular Carcinogenesis, The Netherlands Cancer Institute, Amsterdam;; 9 Division of Clinical Pharmacology, Department of Medicine, University of Cape Town and; 10 Khayelitsha Hospital, Cape Town, South Africa; and; 11 Division of Medicine, Imperial College London, United Kingdom

**Keywords:** HIV, tuberculosis, mortality, innate immunity, mycobacteremia

## Abstract

**Background:**

Case fatality rates among hospitalized patients diagnosed with human immunodeficiency virus (HIV)-associated tuberculosis remain high, and tuberculosis mycobacteremia is common. Our aim was to define the nature of innate immune responses associated with 12-week mortality in this population.

**Methods:**

This prospective cohort study was conducted at Khayelitsha Hospital, Cape Town, South Africa. Hospitalized HIV-infected tuberculosis patients with CD4 counts <350 cells/µL were included; tuberculosis blood cultures were performed in all. Ambulatory HIV-infected patients without active tuberculosis were recruited as controls. Whole blood was stimulated with *Escherichia coli* derived lipopolysaccharide, heat-killed *Streptococcus pneumoniae*, and *Mycobacterium tuberculosis*. Biomarkers of inflammation and sepsis, intracellular (flow cytometry) and secreted cytokines (Luminex), were assessed for associations with 12-week mortality using Cox proportional hazard models. Second, we investigated associations of these immune markers with tuberculosis mycobacteremia.

**Results:**

Sixty patients were included (median CD4 count 53 cells/µL (interquartile range [IQR], 22–132); 16 (27%) died after a median of 12 (IQR, 0–24) days. Thirty-one (52%) grew *M. tuberculosis* on blood culture. Mortality was associated with higher concentrations of procalcitonin, activation of the innate immune system (% CD16+CD14+ monocytes, interleukin-6, tumour necrosis factor-ɑ and colony-stimulating factor 3), and antiinflammatory markers (increased interleukin-1 receptor antagonist and lower monocyte and neutrophil responses to bacterial stimuli). Tuberculosis mycobacteremia was not associated with mortality, nor with biomarkers of sepsis.

**Conclusions:**

Twelve-week mortality was associated with greater pro- and antiinflammatory alterations of the innate immune system, similar to those reported in severe bacterial sepsis.

## Summary

Mortality remains unacceptably high in patients hospitalized with human immunodeficiency virus–associated tuberculosis. Among these patients, half had mycobacteremia. Death was associated with higher concentrations of immune activation and anti-inflammatory markers, and impaired ex vivo innate responses to bacterial antigens.

Tuberculosis remains the most frequent cause of hospitalization and death in human immunodeficiency virus (HIV)-infected patients [1]. Mortality is particularly high among HIV-infected patients who start tuberculosis treatment while in the hospital, ranging from 6% to 32% [2].

The causes of this high mortality (despite receiving tuberculosis treatment) are not well defined. Post-mortem examinations report disseminated tuberculosis as a primary cause of death in many HIV-infected persons [3, 4]. However, tuberculosis mycobacteremia, which is a frequent finding in febrile HIV-infected inpatients [5, 6], has not been consistently associated with mortality [5, 7, 8].

Although there is no published literature to date on immunological changes that accompany mycobacteremia, bacterial sepsis has been studied extensively. Bacterial sepsis is characterized by imbalanced host responses with both proinflammatory and immune suppressive features [9], including monocyte deactivation, neutrophil dysfunction, and increased production of interleukin (IL)-10 [10], potentially contributing to a heightened risk for secondary infections [9, 11].

In HIV-associated tuberculosis (HIV-tuberculosis), similar changes have been described. Reduced innate responses upon stimulation in vitro with lipopolysaccharide (LPS) and heat-killed *Mycobacterium tuberculosis* (*Mtb*) have been associated with death and poor outcome [12], and failure of cellular immune recovery has been associated with death after antiretroviral therapy (ART) initiation in tuberculosis patients [13, 14].

We hypothesized that hospitalized HIV-tuberculosis patients, compared with HIV-infected outpatients without tuberculosis (controls), would have elevation of sepsis biomarkers and proinflammatory cytokines as well as evidence of increased antiinflammatory signalling and decreased innate immune responses to bacterial stimuli and that these features would be associated with mortality. Second, we hypothesized that tuberculosis mycobacteremia would be associated with these immunological features and mortality. Additionally, we tested whether ex vivo treatment with recombinant interferon (IFN)-ɣ (a potential host-directed therapy) could restore monocyte responses to LPS.

## METHODS

### Study Design and Population

We conducted a prospective cohort study in Khayelitsha, Cape Town, South Africa. Khayelitsha has a tuberculosis notification rate of 1065/100 000 person-years (City of Cape Town, unpublished data) and antenatal HIV seroprevalence of 34% [15].

Nonpregnant HIV-infected patients with CD4 counts <350 cells/µL and diagnosed with tuberculosis on admission to Khayelitsha Hospital were recruited between June 2014 and October 2014. Patients were excluded if they were transfused or had received more than 1 dose of tuberculosis treatment within the preceding month. Patients with microbiologically proven rifampicin-susceptible tuberculosis were included. Selection bias was minimized by using a random selection procedure.

HIV-infected outpatients with CD4 counts <350 cells/µL without active tuberculosis were recruited at Úbuntu Clinic, Khayelitsha, Cape Town (controls). To ensure more appropriate matching, only control patients with CD4 counts <150 cells/µL were included in the final analyses.

### Outcomes and Definitions

We aimed to determine immunologic changes associated with the primary outcome of 12-week mortality. Second, we assessed associations of tuberculosis mycobacteremia, defined as at least 1 MycoFlytic blood culture growing *Mtb,* with immunologic profile and outcome. Sepsis definitions were adapted from published criteria (Supplementary Table 1) [9].

### Procedures

A detailed description of ethical aspects and data collection is provided in the Supplementary Methods. All participants had the following tests performed: Xpert MTB/RIF (*Mycobacterium tuberculosis*/rifampin) assay on sputum and urine, tuberculosis culture on sputum, chest X ray, full blood count and differential, chemistry, HIV viral load, and CD4 count. MycoFlytic blood cultures were performed for hospitalized patients. Control patients were excluded if a tuberculosis symptom screen or any tuberculosis diagnostic test was positive. Hospitalized patients were contacted by telephone at 4 weeks and clinically reviewed at 12 weeks.

Whole blood was stimulated for 6 hours. Anti-(myco-)bacterial responses were tested using *Escherichia coli*–derived LPS, heat-killed *Streptococcus pneumoniae*, and heat-killed *Mtb* strain H37Rv. The ex vivo effect of IFN-ɣ was assessed in a costimulation assay with LPS.

Samples were stained with surface and intracellular markers (Supplementary Table 2) and acquired on a BD LSR Fortessa Flow Cytometer. Data were analyzed in FlowJo, version 10 (Ashland, Oregon). Gating strategies are illustrated in Supplementary Figure 1. Concentrations of 12 cytokines (Supplementary Table 2) were measured in culture supernatants using a Luminex multiplex assay.

### Statistical Analyses

Data were analyzed using SPSS, version 22 (IBM, Chicago, Illinois); GraphPad PRISM, version 6 (San Diego, California); and R (Vienna, Austria).

Medians were compared among groups using the appropriate statistical test. Variables were investigated for associations with mortality in a Cox proportional hazards model. A priori–defined potential confounders were age, sex, ART status, HIV viral load and CD4 count, and Luminex plate number; a variable was retained in the model if introduction led to a >10% change in the effect measure.

All reported *q* values were calculated using Benjamini-Hochberg procedures for multiple-testing correction [16]. *P* values < .05 and *q* values <.10 were regarded as significant.

Patients for whom data were available for all stimulation conditions were included (n = 37) for principal component analysis (PCA); 90 variables were included per patient. Using a Shapiro test, all variables with *P* < .05 were log-transformed, and scaling was done. Data missing due to technical difficulties (112/3300 values; 3.4%) were imputed using the K-nearest-neighbor technique.

## RESULTS

### Participants

Of 124 HIV-infected patients with probable tuberculosis who were enrolled, 60 participants with confirmed rifampicin-susceptible tuberculosis were included in this analysis. Twenty-seven HIV-infected control patients were included (Supplementary Figure 2). All HIV-tuberculosis patients fulfilled criteria for sepsis; 38/60 (63.3%) had severe sepsis and 23/60 (38.3%) had septic shock (Table 1). Tuberculosis treatment was initiated in all patients a median of 1 day (interquartile range [IQR], 0.5–2.5 days) after recruitment. Co-trimoxazole prophylaxis was initiated in the hospital in 28 (46.7%) patients, and 54 (90.0%) received broad-spectrum antibiotics (mostly ceftriaxone). Most patients were on ART at enrollment (31/60, 52%; Table 1).

**Table 1.** Clinical Characteristics and Hematology and Chemistry Results

Variables	**HIV + Tuberculosis Controls (n** = **14**)	**HIV + Tuberculosis Patients (n** = **60**)	***q* Value** ^**a**^	**Survivors (n** = **44**)	**Deceased (n** = **16**)	***q* Value** ^**b**^
Demographics						
Male sex (n, %)	4/14 (29)	30/60 (50)	0.21	24/44 (55)	6/16 (38)	0.22
Age	34 (29–42)	39 (33–45)	0.25	36 (31–41)	44 (35–55)	**0.09**
ART status (n, %)						
Naive	8/14 (57)	31/60 (52)	0.79	25/43 (57)	6/16 (38)	0.35
On ART	1/14 (7)	16/60 (27)		11/43 (25)	5/16 (31)	
Defaulted	5/14 (36)	12/60 (20)		7/43 (16)	5/16 (31)	
HIV disease markers						
CD4 count (cells/µL)	71 (56–121)	53 (22–132)	0.26	57 (22–139)	45 (19–90)	0.63
HIV VL (log)	4.55 (2.16–5.29)	5.62 (3.97–6.08)	**0.02**	5.68 (4.85–6.16)	4.39 (3.18–5.69)	0.17
HIV VL undetectable (n, %)	3 (21)	5 (8.3)	0.19	3 (6.8)	2 (12.5)	0.48
Tuberculosis diagnostics						
Sputum culture/Xpert positive (n, %)	0/14 (0)	35/41 (85)	**0.0002**	26/31 (84)	9/10 (90)	0.69
Urine Xpert positive (n, %)	0/14 (0)	15/35 (43)	**0.0002**	9/26 (35)	6/9 (66)	0.22
Tuberculosis blood culture positive (n, %)	ND	31/60 (52)		24/44 (55)	7/16 (44)	0.60
Sepsis criteria						
Sepsis (n, %)	0/14 (0)	60/60 (100)	**0.0002**	44/44 (100)	16/16 (100)	ND
Severe sepsis (n, %)	0/14 (0)	39/60 (65)	**0.0002**	26/44 (59.1)	13/16 (81.3)	0.22
Septic shock (n, %)	0/14 (0)	23/60 (38)	**0.0002**	14/44 (31.8)	9/16 (56.3)	0.22
Hematology						
Hemoglobin (g/dL)	12.0 (10.1–12.9)	8.9 (6.7–10.8)	**0.002**	9.3 (7.0–11.4)	6.9 (6.4–9.8)	0.22
White cell count (10^9^/L)	3.7 (2.9–4.5)	6.4 (4.4–9.4)	**0.0002**	7.0 (4.8–10.4)	5.8 (3.9–7.6)	0.22
Neutrophils (10^9^/L)	1.2 (1.3–2.5)	5.8 (3.6–9.3)	**0.0002**	6.0 (4.2–9.5)	4.7 (2.7–6.8)	0.22
Lymphocytes (10^9^/L)	1.00 (0.88–1.43)	0.56 (0.36–0.96)	**0.002**	0.58 (0.37–1.03)	0.49 (0.29–0.78)	0.31
Monocytes (10^9^/L)	0.36 (0.33–0.42)	0.33 (0.16–0.51)	0.53	0.39 (0.19–0.58)	0.20 (0.12–0.43)	0.22
Platelets (10^9^/L)	220 (192–316)	251 (179–325)	0.75	250 (183–325)	259 (119–330)	0.78
Serum chemistry						
Glucose (mmol/L)	ND	5.3 (4.9–6.5)	ND	5.3 (5.0–6.2)	5.5 (3.9–7.8)	0.86
Lactate (mmol/L)	ND	1.9 (1.3–3.0)	ND	1.8 (1.2–2.6)	2.7 (1.5–3.8)	0.22
Procalcitonin (μg/L)	ND	2.42 (0.54–8.67)	ND	1.31 (0.36–4.98)	8.28 (3.63–61.05)	**0.07**
C-reactive protein (mg/L)	ND	143 (94–191)	ND	139 (83–191)	143 (129–230)	0.39
Albumin (g/L)	ND	23 (19–28)	ND	25 (19–28)	20 (17–24)	0.15
Creatinine (µmol/L)	ND	90 (49–131)	ND	84 (60–139)	105 (61–195)	0.63

χ^2^ tests were used for categorical data, Student *t* test for normally distributed continuous data, and Mann-Whitney *U* tests for nonnormally distributed data. Medians and interquartile ranges are shown for all variables, unless otherwise indicated.

Abbreviations: ART, antiretroviral therapy; HIV, human immunodeficiency virus; ND, not determined; VL, viral load.

^a^ *q* value of comparisons between the HIV-infected control group and HIV-associated tuberculosis patients. Significant differences (*q* < .10) are in bold.

^**b**^ *q* value of comparisons between the group of patients who died and the group who survived. Significant differences (*q* < .10) are in bold.

### Clinical Presentation and Outcomes

Compared with controls, HIV-tuberculosis patients had higher HIV viral loads and were more often anaemic (Table 1). Also, 35/41 (85%) HIV-tuberculosis patients who could produce sputum had a positive sputum Xpert MTB/RIF or culture for *Mtb*; 31/60 (52%) patients grew *Mtb* on blood culture; 15/35 (43%) had a positive urine Xpert MTB/RIF; and 16 patients had positive cultures or Xpert MTB/RIF from other sites. In addition, 28/60 (47%) patients had standard bacterial blood cultures performed prior to receipt of broad-spectrum antibiotics; none grew pathologic bacteria. By 12 weeks, 16 HIV-tuberculosis patients had died (26.7%) a median of 12 (IQR, 0–24) days after enrollment (Supplementary Table 3); none were lost to follow-up. All deaths occurred while the patients were in the hospital. Four patients had suspected bacterial sepsis when they died, and 11 deaths were attributed to disseminated tuberculosis; 1 of them had disseminated tuberculosis and features that suggested bacterial sepsis.

Compared with HIV-tuberculosis patients who survived, patients who died were older and presented more often with severe sepsis or septic shock (Table 1). Mycobacteremia was not associated with mortality (crude hazard ratio [cHR], 0.74; 95% confidence interval [CI], 0.3–2.0; and *P* = .55; adjusted HR [aHR], 0.80; 95% CI, 0.3–2.2; and *P* = .64), nor was time to tuberculosis treatment in days (cHR, 0.87; 95% CI, 0.69–1.11; and *P* = .26; aHR, 0.82; 95% CI, 0.63–1.07; and *P* = .14). HIV-tuberculosis patients who died had significantly higher concentrations of procalcitonin compared with survivors (Table 1).

### Immune Phenotype in HIV-Tuberculosis Versus Controls

**Figure 1.**
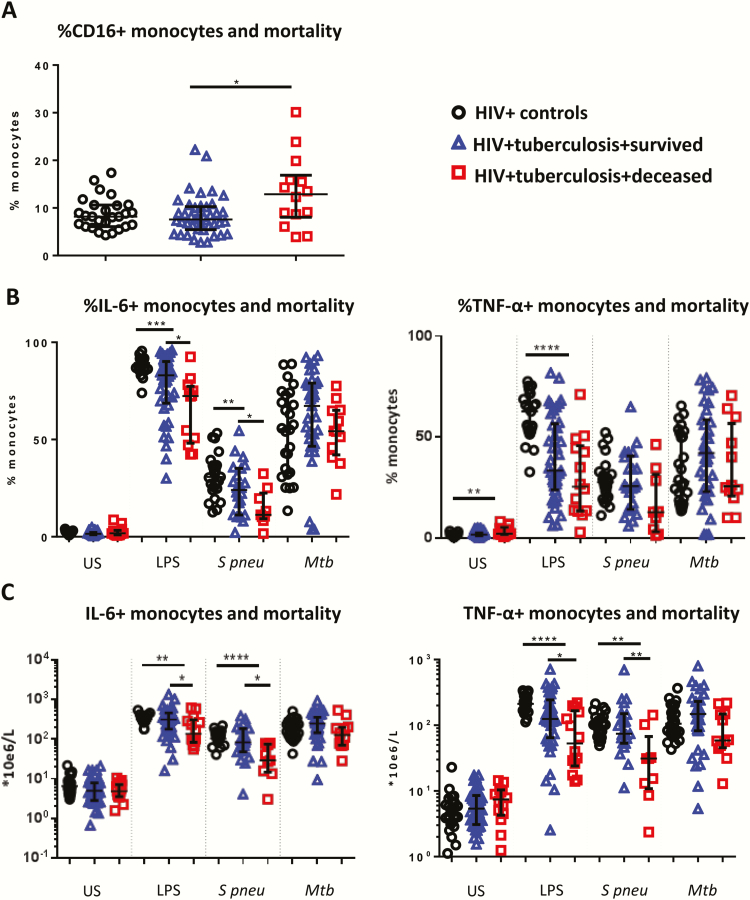
Monocyte responses and mortality. Median values and interquartile ranges are shown for the percentage of CD16+ monocytes in unstimulated samples (human immunodeficiency virus [HIV] controls, n = 26; HIV-tuberculosis patients, n = 55) (*A*), and percentages (*B*) and absolute counts (*C*) of interleukin-6+ and tumor necrosis factor-ɑ+ monocytes in response to lipopolysaccharide (HIV controls, n = 25; HIV-tuberculosis patients, n = 55), *Streptococcus pneumoniae* (HIV controls, n = 25; HIV-tuberculosis patients, n = 31), and *Mycobacterium tuberculosis* (HIV controls, n = 26; HIV-tuberculosis patients, n = 47) respective stimulants. Absolute counts were derived by multiplying the percentage of positive cells by the monocyte count obtained from the National Health Laboratory Service clinical laboratory. HIV-infected control patients (black circles), HIV-tuberculosis patients who survived (blue triangles), and HIV-tuberculosis patients who died (red squares) are shown. Kruskal-Wallis and Mann-Whitney *U* tests were used for comparisons between groups. *P < .05, **P < .01, *** *P* < .001, ****P < .0001.

Abbreviations: HIV, human immunodeficiency virus; IL, interleukin; LPS, lipopolysaccharide; *Mtb*, heat-killed *Mycobacterium tuberculosis*; *S. pneumoniae*, *Streptococcus pneumoniae*; TNF, tumour necrosis factor; US, unstimulated.

In unstimulated samples, patients with HIV-tuberculosis had higher percentages of tumor necrosis factor (TNF)-α+ monocytes compared with controls (Figure 1) and higher supernatant concentrations of colony-stimulating factor (CSF)-3 and IFN-ɣ, whereas supernatant concentrations of IL-1β and IL-8 were lower (Table 2).

**Table 2.** Cytokine Concentrations in Culture Supernatants

	**HIV + Tuberculosis Controls**		**HIV + Tuberculosis Patients**		***q* Value** ^**a**^	**HIV + Tuberculosis Deceased**		**HIV + Tuberculosis Survived**		***q* Value** ^**b**^
**Cytokine**	**Median**	**IQR**	**Median**	**IQR**		**Median**	**IQR**	**Median**	**IQR**	
**Unstimulated**	**n** = **14**		**n** = **60**			**n** = **16**		**n** = **44**		
CSF-3	**51**	**39.78–86.03**	**77**	**52.4–163.5**	**0.04**	**134.7**	**75.3–341.8**	**66.86**	**48.6–143.9**	**0.09**
CSF-2	14	10.65–18.82	12	10.5–13.7	0.30	11.69	10.7–14.2	12.01	10.4–13.7	1.00
IFN-A2	15	10.97–20.00	14	10.9–18.4	0.66	13.41	11.7–18.1	14.07	10.1–19.1	0.99
IFN-ɣ	**45**	**16.36–74.39**	**92**	**39.7–249.4**	**0.02**	168.5	81.5–246.6	72.73	35.1–269.6	0.49
IL-10	23	14.77–34.57	24	14.9–29.6	0.96	25.91	14.4–62.6	23.11	14.9–27.7	0.61
IL-12p40	21	13.84–31.33	20	16.3–23.7	0.82	21.03	14.7–27.0	19.57	16.3–23.5	0.76
IL-1RA	151	83.78–212.6	252	109.2–495.3	0.14	566.7	130.2–958.6	241.9	107.2–423.8	0.30
IL-1β	**6**	**3.75–29.79**	**3**	**2.4–4.7**	**0.01**	3.19	2.5–4.4	3.14	2.34–5.0	0.93
IL-6	91	34.59–442.9	50	24.4–106.8	0.16	55	41.3–128.2	45.09	20.4–90.8	0.35
IL-8	**1826**	**922.5–4095**	**619**	**276.8–1563**	**0.01**	579.4	279–1314	658.8	220.1–1781	0.89
TNF-α	111	69.52–291.7	93	62.7–153.9	0.48	125.1	72.7–202	88.18	54.9–137.2	0.30
LPS	**n** = **14**		**n** = **60**			**n** = **16**		**n** = **44**		
CSF-3	363	259.5–514.8	393	206.1–614.0	0.89	439.5	227.6–1235	370.6	202.8–557.3	0.66
CSF-2	**18**	**12.46–23.33**	**13**	**11.4–14.8**	**0.02**	12.69	11.4–15.4	13.28	11.4–14.8	0.79
IFN-A2	16	14.30–24.19	15	11.9–19.1	0.31	14.63	11.9–17.6	14.94	11.7–20.8	0.93
IFN-ɣ	**38**	**29.85–74**	**87**	**51.3–217.4**	**0.02**	160.2	83.6–229.4	76.97	44.4–212	0.31
IL-10	236	199.9–493	225	83.2–445.2	0.50	170.9	65.4–341.1	236	112.9–635.3	0.31
IL-12p40	**130**	**63.49–274**	**38**	**25.2–82.8**	**0.01**	33.82	23.5–71.5	42.07	25.8–89.0	0.61
IL-1RA	**768**	**388.5–1511**	**2219**	**993.7–4478**	**0.01**	1961	500–4498	2302	1137–4607	0.66
IL-1β	**2740**	**1772–4494**	**337**	**61.2–1024**	**0.0001**	149.6	43.3–451	416.3	89.3–1102	0.24
IL-6	**10432**	**3015–12118**	**7860**	**2664–9437**	**0.07**	2941	1829–9386	8372	3442–9475	0.31
IL-8	7229	3150–9331	4079	1788–9546	0.29	3229	1778–9309	4891	1793–9684	0.76
TNF-α	**7452**	**3634–10339**	**1669**	**823.4–4490**	**0.01**	1197	589.6–2734	2506	961.6–5070	0.21
*Streptococcus pneumoniae*	**n** = **14**		**n** = **37**			**n** = **11**		**n** = **26**		
CSF-3	**81**	**65–122**	**149**	**88.1–240.6**	**0.04**	**237.5**	**138.0–483.5**	**103**	**78.1–171**	**0.08**
CSF-2	**21**	**14–27**	**13**	**11.4–14.8**	**0.0001**	12.69	9.7–13.3	12.69	11.5–14.9	0.76
IFN-A2	16	12--21	14	11.5–19.4	0.50	13.76	12.5–22.5	13.64	11.0–18.6	0.81
IFN-ɣ	**35**	**25–82**	**118**	**49.6–211.0**	**0.02**	150.3	56.5–182.5	97.6	45.9–224.3	0.80
IL-10	39	32–102	41	25.3–72.2	0.77	34.88	21.3–76.8	42.75	25.7–63.8	0.89
IL-12p40	**43**	**27–60**	**25**	**19.9–35.0**	**0.04**	25.44	18.2–30.0	25.53	20.7–37.7	0.76
IL-1RA	**422**	**168–488**	**801**	**499.3–1880**	**0.0001**	1030	424.2–3361	786.9	522.3–1789	0.76
IL-1β	**955**	**786–1252**	**131**	**21.2–265.6**	**0.0001**	**20.55**	**9.3–61.5**	**152**	**47.7–311.5**	**0.08**
IL-6	**7491**	**2114–8801**	**1581**	**339.5–3673**	**0.01**	**834.6**	**169.6–1586**	**2031**	**409.5–4748**	**0.08**
IL-8	10000	3544–12138	9067	2890–11003	0.52	4028	1394–10033	9415	4394–11286	0.35
TNF-α	**5158**	**2965–7742**	**1270**	**480.0–3063**	**0.01**	488.2	248.8–1021	2074	750.7–3263	0.20
*Mycobacterium tuberculosis*	**n** = **14**		**n** = **54**			**n** = **14**		**n** = **40**		
CSF-3	**107**	**64–275**	**404**	**178.4–902.1**	**0.01**	383.1	305.9–924.2	445	135.4–929.1	0.89
CSF-2	32	23–45	21	13.6–32.3	0.10	17.73	12.7–20.8	23.61	14.4–39.3	0.21
IFN-A2	16	13–21	15	12.2–19.9	0.42	14.6	12.0–19.2	14.74	12.3–20.5	0.89
IFN-ɣ	**37**	**18–68**	**138**	**55.2–289.0**	**0.01**	149.7	62.2–226.1	119.2	54.5–293.3	0.96
IL-10	125	61–194	93	47.4–306.9	0.93	54.45	44.2–148.4	104.8	62.9–365.8	0.30
IL-12p40	28	17–42	25	17.5–37.2	0.89	22.32	16.3–30.1	25.94	18.1–38.7	0.49
IL-1RA	**295**	**139–554**	**844**	**315.7–1517**	**0.01**	1051	192.9–2238	816.9	369.9–1448	0.96
IL-1β	705	389–1305	415	48.6–1203	0.48	202.9	29.6–411.2	686.4	67.8–2329	0.19
IL-6	5744	2441–7976	5848	1528–10229	0.77	1972	1097–8467	7954	2044–10663	0.26
IL-8	9121	2805–11520	9303	3516–11278	0.73	8351	5566–10254	9798	3430–11789	0.66
TNF-α	2103	1508–3971	2296	755.0–6655	0.74	1153	548.6–2473	2793	844.6–8411	0.20

Median and IQRs of cytokine concentrations measured in culture supernatants of HIV-infected control patients, HIV-tuberculosis survivors, and HIV-tuberculosis nonsurvivors, respectively. Values are in picogram per milliliter. Mann-Whitney *U* tests were used for nonparametric data and Student *T* tests for parametric data.

Abbreviations: CSF, colony-stimulating factor; HIV, human immunodeficiency virus; IFN, interferon; IL, interleukin; IQR, interquartile range; LPS, lipopolysaccharide; TNF, tumour necrosis factor.

^a^ *q* value of comparisons between the HIV-infected control group and HIV-associated tuberculosis patients. Significant differences (q < .10) are in bold.

^b^ *q* value of comparisons between the group of patients who died and the group of patients who survived. Significant differences (q < .10) are in bold.

Whole blood of HIV-tuberculosis patients showed a significantly reduced production of proinflammatory cytokines following stimulation compared with controls (Figure 1, Table 2). In response to LPS, percentages of IL-6+ and TNF-α+ monocytes were lower (Figure 1), as were percentages of IL-6+ and TNF-α+ neutrophils (*q* = 0.003, *q* = 0.003, respectively) and supernatant concentrations of CSF-2 and proinflammatory cytokines IL-12p40, IL-1β, IL-6, IL-8, and TNF-α (Table 2). Supernatant IFN-ɣ and antiinflammatory IL1-RA concentrations were higher in HIV-tuberculosis patients (Table 2).

In *S. pneumoniae* stimulations, percentages of IL-6+ and TNF-α+ monocyte counts (*q* = 0.003) and IL-6+ absolute monocyte (*q* = 0.003) counts were lower in HIV-tuberculosis patients (Figure 1), as were percentages and absolute counts of IL-6+ (*q* = 0.003) and TNF-α+ (*q* = 0.01) neutrophils. HIV-tuberculosis patients had higher supernatant concentrations of CSF-3, IFN-ɣ, and IL-1-receptor-antagonist (IL-1RA) and lower concentrations of CSF-2, IL-12p40, IL-1β, IL-6, and TNF-α (Table 2).

In *Mtb* stimulations, percentages and absolute counts of IL-6+ and TNF-α+ monocytes were similar in HIV-tuberculosis patients and controls (Figure 1). Percentages of IL-6+ and TNF-α+ neutrophils were lower in HIV-tuberculosis patients (*q* = 0.01 and *q* = 0.07, respectively), whereas concentrations of CSF-3, IFN-ɣ, and IL-1RA were higher (Table 2).

### Pro- and Antiinflammatory Changes in Hospitalized HIV-Tuberculosis Patients Who Died Versus Survivors

**Figure 2.**
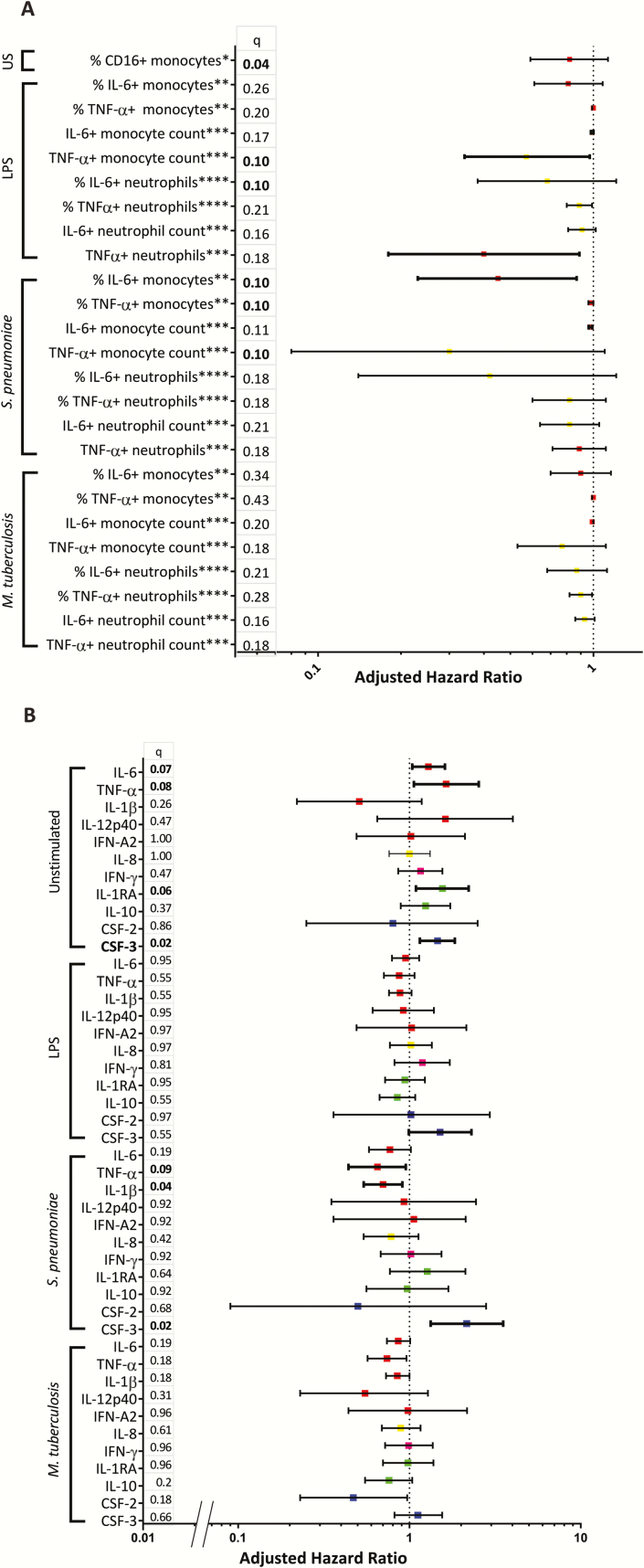
Death hazard ratios for intra- and extracellular cytokines. *A,* Adjusted hazard ratios (aHR) for monocyte activation and intracellular cytokines measured in monocytes and neutrophils. Monocyte responses are in red; neutrophil responses are in yellow. *per 1% increase; ** per 10% increase; *** per 10 ^6^/L increase; **** per 0.1% increase. *B,* The aHRs for the extracellular cytokines measured. HRs are per log_2_ pg/mL increase. Shown in red are proinflammatory cytokines mainly produced by monocytes; in yellow are proinflammatory cytokines mainly related to neutrophil function; in purple are pro-inflammatory cytokines related to T helper 1 function; in green are antiinflammatory cytokines; in dark blue are growth factors. All HRs are adjusted for age, sex, CD4 count, HIV viral load, antiretroviral therapy status, and plate number, where applicable. *q* values are shown on the left; significant associations are in bold. Abbreviations: CSF, colony-stimulating factor; IFN, interferon; IL, interleukin; LPS, lipopolysaccharide; TNF, tumour necrosis factor; US, unstimulated.

Immune activation was associated with mortality. Patients who died had higher percentages of CD16+CD14+ monocytes and higher supernatant concentrations of CSF-3 in unstimulated samples compared with patients who survived (Figure 1, Table 2). Higher percentages of activated CD16+CD14+ monocytes (Figure 2A) were independently associated with mortality, as were supernatant concentrations of CSF-3, IL-1RA, IL-6, and TNF-α (Figure 2).

**Figure 3.**
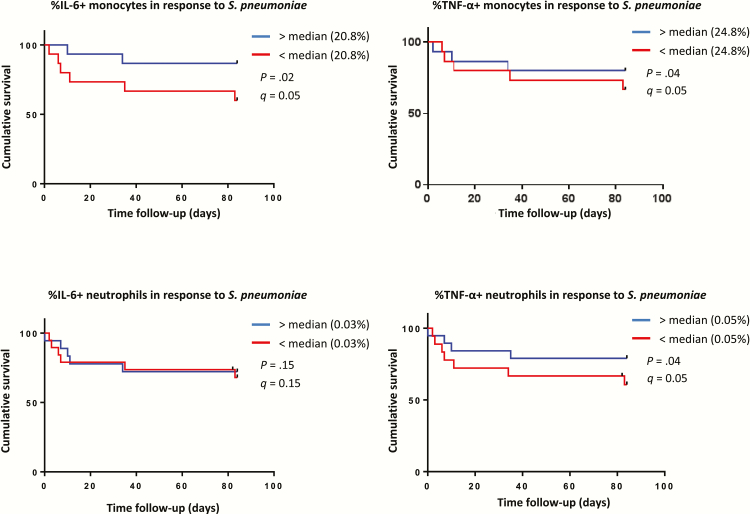
Time to death and innate responses to *Streptococcus pneumonia.* Kaplan-Meier curves for the survival analysis of monocyte (n = 31) and neutrophil (n = 37) production of interleukin-6 and tumor necrosis factor-ɑ in response to *S. pneumoniae* are shown. The population of patients with human immunodeficiency virus–associated tuberculosis was split at the median for each respective analysis. Blue lines are for the group with cytokine production above the median; in red, the patients who had responses below the median. Abbreviations: IL, interleukin; TNF, tumour necrosis factor.

Compared with survivors, patients who died had lower absolute counts of IL-6+ and TNF-α+ producing monocytes in response to LPS and *S. pneumoniae* (Figure 1). Lower supernatant IL-1β and IL-6 concentrations and higher CSF-3 concentrations were measured in *S. pneumoniae* stimulations of patients who died (Table 2). Impaired monocyte and TNF-α and IL-6 production in response to *S. pneumonia*e and lower percentages of IL-6+ neutrophils in response to LPS were independently associated with mortality (Figure 2A, Figure 3), as were increased supernatant concentrations of CSF-3 and lower concentrations of IL-1β and TNF-ɑ in response to *S. pneumoniae* (Figure 2B).

In response to heat-killed *Mtb*, patients who died had lower absolute counts of IL-6+ and TNF-ɑ+ neutrophils. No differences were seen in *Mtb* culture supernatants (Table 2). *Mtb* responses were not associated with mortality (Figure 2).

**Figure 4.**
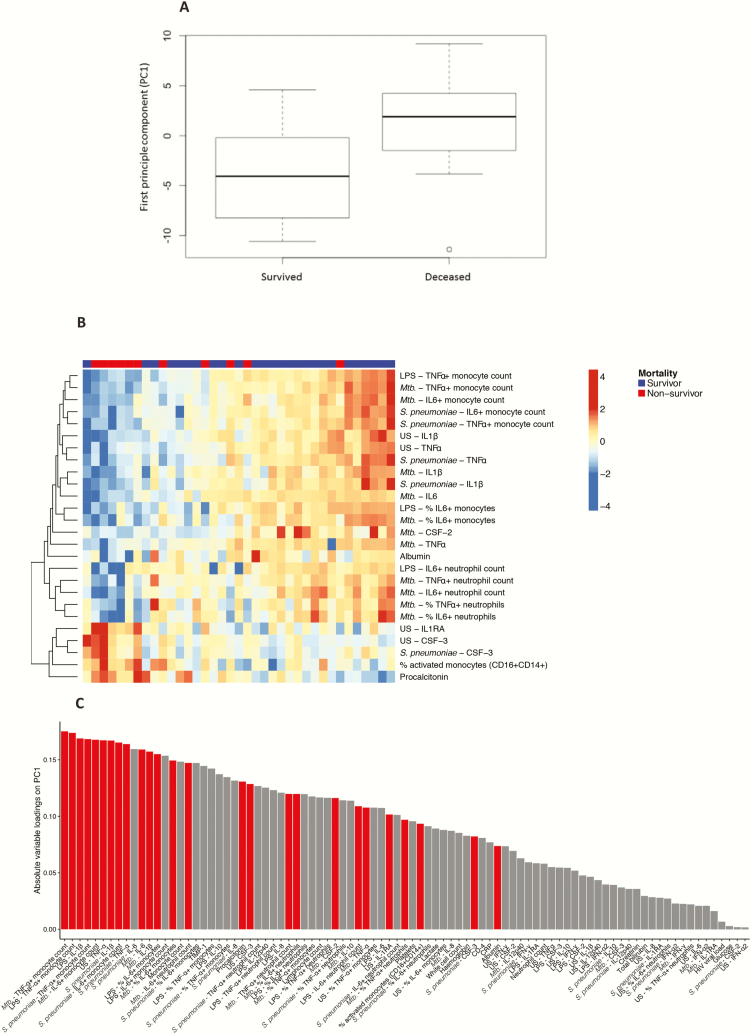
Principal component analysis. *A,* Values for principal component 1 (PC1) for patients with human immunodeficiency virus–associated tuberculosis who died vs those who survived; patients who died had significantly higher values for PC1. B, Loadings of respective variables on PC1; variables significantly associated with clinical outcome are red. Variables significantly associated with clinical outcome (Mann-Whitney *U* test *q* < .10 and *P* < .05) tend to have high loadings, hence, contribute strongly to PC1. *C,* Heat map showing variables associated significantly with mortality in principal component analysis. Survivors are shown in blue, nonsurvivors in red. Increased production of proinflammatory cytokines in unstimulated samples appeared to be associated with mortality, as well as impaired production of proinflammatory cytokines in response to all antigen stimuli used. Abbreviations: CSF-3, CSF-2, colony-stimulating factor; IL, interleukin; LPS, lipopolysaccharide; Mtb, heat-killed *Mycobacterium tuberculosis*; *S. pneumoniae*, *Streptococcus pneumoniae*; TNF, tumour necrosis factor; US, unstimulated.

In PCA, the first principal component (PC1) was significantly associated with mortality (*P* = .003; Figure 4A–B). After all samples were arranged by their value on PC1, an immunological signature associated with mortality was revealed (Figure 4C), characterized by increased production of cytokines in unstimulated conditions and impaired production of proinflammatory cytokines in response to all antigen stimuli.

### *Mtb* Mycobacteremia Is Associated With Cytopenia but Not With Sepsis Severity

Mycobacteremic patients had lower platelet (median 203 vs 311 × 10^9^/L), lymphocyte (median 0.42 vs 0.66 × 10^9^/L), and monocyte counts (median 0.25 vs 0.42 × 10^9^/L) compared with non-mycobacteremic patients (*P* = .05, *P* = .0003, and *P* = .03, respectively). There were no differences in plasma concentrations of procalcitonin or lactate, percentages of CD16+CD14+ monocytes in unstimulated samples, or percentages of IL-6+ or TNF-α+ monocytes in response to stimulations (Supplementary Figure 3). Mycobacteremic patients had lower absolute counts of IL-6+ monocytes compared with non-mycobacteremic tuberculosis patients in unstimulated samples and lower absolute counts of both IL-6+ and TNF-α+ monocytes in response to LPS (Supplementary Figure 3). There were no differences in supernatant cytokine concentrations (Supplementary Table 4). None of the variables were associated with mycobacterial load, expressed in days to blood culture positivity (results not shown).

### No Reversal of Immune Deficits With IFN-ɣ Costimulation

There were no differences in neutrophil and monocyte capacity to produce IL-6 or TNF-ɑ, nor in the concentration of any of the extracellular cytokines, when the LPS with IFN-ɣ costimulation was compared with LPS only.

## DISCUSSION

In HIV-infected patients diagnosed while in the hospital with microbiologically proven drug-susceptible tuberculosis, 12-week mortality was 27%. More than half of patients had mycobacteremia; however, this was not associated with mortality. Patients who died had an immune phenotype characterized by higher concentrations of proinflammatory cytokines (CSF-3, TNF-ɑ, and IL-6) and antiinflammatory IL-1RA, an increased proportion of proinflammatory CD14+CD16+ monocytes, and impaired capacity of innate cells to produce proinflammatory cytokines in response to bacterial antigen stimuli.

Increased concentrations of proinflammatory cytokines CSF-3, IL-6, and TNF-ɑ in unstimulated samples were associated with mortality. These cytokines are mainly produced by innate cells, suggesting a more activated state of the innate immune system in patients who die. This is consistent with results from other studies of mortality in HIV-associated tuberculosis [14], ART-naive patients who start ART [17], and bacterial sepsis in HIV-infected patients [18, 19]. There are several plausible explanations for this association. It is possible that immune activation reflects more disseminated tuberculosis. Another possible explanation is a causal relation: higher concentrations of proinflammatory cytokines led to increased tissue damage, organ failure, and immune exhaustion, impairing host defense to other pathogens.

Increased percentages of CD16+CD14+ monocytes were associated with mortality. This activated monocyte subset generally produces higher amounts of proinflammatory cytokines and is more phagocytic compared with the classic CD16 subset [20]. However, previous studies have shown that monocyte functionality may be impaired in active tuberculosis, with CD16+CD14+ monocytes refractory to dendritic cell maturation, leading to impaired antigen presentation and decreased secretion of IL-1β and IL-12 [21, 22]. Impaired phagocytic and antigen-presenting capacity of CD14+CD16+ cells has also been described in bacterial sepsis. Monocyte deactivation, with reduced production of TNF-ɑ in response to LPS was associated with fatal outcome [10, 23]. Our findings indicate that expansion of the CD16+ CD14+ monocyte population is observed together with impaired total monocyte and neutrophil responses to bacterial antigens in patients who die, potentially contributing to mortality.

The immunological phenotype associated with mortality is similar to what has been described in bacterial sepsis. In bacterial sepsis, time to intravenous broad-spectrum antibiotic treatment is associated inversely with survival [9]. We did not find such an association for time to tuberculosis treatment. Although a potential association might have been masked by the lack of exact data on time of tuberculosis treatment initiation in hours, a more likely explanation is the fact that tuberculosis treatment is administered orally, and drug absorption might be hampered by intestinal tissue damage by tuberculosis or HIV.

Although invasive pneumococcal disease is one of the most frequent and lethal bacterial infections in HIV-infected patients, most studies focus on LPS responses. Interestingly, we found that impaired responses to *S. pneumoniae* were also strongly associated with mortality, indicating defects in host defense to this pathogen.

Reduced proinflammatory responses of innate cells to LPS and *S. pneumoniae* in HIV-tuberculosis patients, compared with controls, suggest that tuberculosis has an immunosuppressive effect that is additive to HIV. CD4 count and HIV viral load were not associated with mortality, whereas the innate immune features were independently associated.

Tuberculosis mycobacteremia was neither associated with mortality nor with more severe derangement of sepsis biomarkers. Mycobacteremia was associated with cytopenia, but there were no functional differences of innate cells. Previous studies have shown that in hospitalized febrile patients, mycobacteremia was associated with mortality [5, 6, 24], whereas in other studies among HIV-tuberculosis patients with mycobacteremia, it was not [7, 8]. Our findings illustrate that patients with severe HIV-tuberculosis can develop features of a sepsis syndrome even when mycobacterial blood cultures are negative.

There is increasing interest in host-directed immunotherapies for tuberculosis [25]. Recombinant IFN-ɣ has been shown to be beneficial in the treatment of cryptococcal meningitis [26] and other fungal infections [27], and a trial to investigate its application for bacterial sepsis is ongoing [28]. We found no effect of ex vivo costimulation with recombinant IFN-ɣ in restoring monocyte responses to LPS. Although ex vivo data cannot be directly extrapolated to in vivo conditions, our findings do not support investigation of recombinant IFN-ɣ as a potential immunotherapy in this patient subset. Our data are supported by 2 clinical trials [25, 29] that showed no beneficial effects of IFN-ɣ on sputum culture conversion in drug-sensitive or drug-resistant pulmonary tuberculosis. CSF-3 was investigated as adjunctive immune therapy for bacterial sepsis, but there was no significant survival benefit [30]. Our data of increased, rather than decreased, concentrations of CSF-3 in patients who die do not support investigation of CSF-3 for host-directed therapy in HIV-tuberculosis.

The early mortality [2] and prevalence of mycobacteremia [6, 7, 24] reported here are similar to that found in studies from Africa and suggest our immunological findings are generalizable to other settings with a high tuberculosis and HIV burden. Limitations of our study include a limited number of bacterial blood cultures and lack of post-mortem examinations. Due to the strategy of treating patients with sepsis syndrome with broad-spectrum antibiotics at primary care referral centers upon referral to the hospital, only 47% of patients had bacterial blood cultures performed prior to receipt of antibiotics. Post-mortem examinations were planned; however, in this study, none of the families agreed to this. Ex vivo markers of immune exhaustion were not measured, and this should be the subject of future studies.

Major strengths of our study are the variety of antigens/organisms used for stimulations, enabling in vitro simulation of gram-negative, gram-positive, and mycobacterial infections. The inclusion of an HIV-infected control group without active tuberculosis and the exclusion of patients without microbiologically confirmed tuberculosis facilitate conclusions on the associations of findings with severe tuberculosis specifically, minimizing misclassification bias of other diagnoses or advanced HIV alone.

Our study has several implications for clinical care and future research. The immune profile observed in HIV-tuberculosis patients, particularly those who died, suggests that disseminated tuberculosis in the context of advanced HIV infection can significantly impair host innate responses to bacteria, possibly resulting in an immunological predisposition to bacterial superinfections [3, 4]. This study adds to our understanding of immunopathology in HIV-tuberculosis. By focusing on patients who required hospital admission and innate immune changes, we confirmed that the high mortality in this patient subset is associated with an immunological phenotype similar to bacterial sepsis. Immune activation, with higher concentrations of proinflammatory cytokines and expansion of the CD16+CD14+ monocyte population, is potentially leading to increased tissue damage. Simultaneously, there is impairment of innate immune functional responses, with reduced production of proinflammatory cytokines in response to antigens of bacterial pathogens. In the future, immunomodulatory interventions that have proven beneficial in bacterial sepsis should also be evaluated for patients with severe HIV-tuberculosis.

## Supplementary Data

Supplementary materials are available at *Clinical Infectious Diseases* online. Consisting of data provided by the authors to benefit the reader, the posted materials are not copyedited and are the sole responsibility of the authors, so questions or comments should be addressed to the corresponding author.

## Supplementary Material

Suppl_Fig_1

Suppl_Fig_2

Suppl_Figure_3

Supplementary_Table_1

Supplementary_Table_2

Supplementary_Table_3

Supplementary_Table_4

Paper_mycobacteremia_OnlineDataSuppl_CID_acc_changes

## Notes

***Acknowledgments.*** The authors thank Rene Goliath, Amanda Jackson, Vanessa January and M.K. Mpalali, Bekekili Kwasa, Lebo Tsekela, and Nonceba Gobe from the University of Cape Town, and Jonathan Ellis and Susan George for their contributions to data collection. We thank Felix Dube for his assistance with the *S. pneumoniae* strains and Bruno Andrade for his assistance with analyzing the Luminex data.

***Financial support.*** This work was supported by the Wellcome Trust (098316). S. J. was supported by grants of the Marie Curie People program and the Studiefonds Ketel 1.

***Potential conflicts of interest:*** All authors: No reported conflicts of interest. All authors have submitted the ICMJE Form for Disclosure of Potential Conflicts of Interest. Conflicts that the editors consider relevant to the content of the manuscript have been disclosed.

## References

[1] Ford N Shubber Z Meintjes G Causes of hospital admission among people living with HIV worldwide: a systematic review and meta-analysis. Lancet HIV 2015; 2:e438–44. 26423651 10.1016/S2352-3018(15)00137-X

[2] Odone A Amadasi S White RG Cohen T Grant AD Houben RM The impact of antiretroviral therapy on mortality in HIV positive people during tuberculosis treatment: a systematic review and meta-analysis. PLoS One 2014; 9:e112017. 25391135 10.1371/journal.pone.0112017 PMC4229142

[3] Ansari NA Kombe AH Kenyon TA Pathology and causes of death in a group of 128 predominantly HIV-positive patients in Botswana, 1997–1998. Int J Tub Lung Dis 2002; 6:55–63. 11931402

[4] Wong EB Omar T Setlhako GJ Causes of death on antiretroviral therapy: a post-mortem study from South Africa. PLoS One 2012; 7:e47542. 23094059 10.1371/journal.pone.0047542 PMC3472995

[5] Crump JA Ramadhani HO Morrissey AB Bacteremic disseminated tuberculosis in sub-Saharan Africa: a prospective cohort study. Clin Infect Dis 2012; 55:242–50. 22511551 10.1093/cid/cis409 PMC3491770

[6] Jacob ST Pavlinac PB Nakiyingi L *Mycobacterium tuberculosis* bacteremia in a cohort of HIV-infected patients hospitalized with severe sepsis in Uganda—high frequency, low clinical suspicion [corrected] and derivation of a clinical prediction score. PLoS One 2013; 8:e70305. 23940557 10.1371/journal.pone.0070305 PMC3734073

[7] Crump JA Wu X Kendall MA Predictors and outcomes of *Mycobacterium tuberculosis* bacteremia among patients with HIV and tuberculosis co-infection enrolled in the ACTG A5221 STRIDE study. BMC Infect Dis 2015; 15:12. 25582793 10.1186/s12879-014-0735-5 PMC4297427

[8] Nakiyingi L Ssengooba W Nakanjako D Predictors and outcomes of mycobacteremia among HIV-infected smear-negative presumptive tuberculosis patients in Uganda. BMC Infect Dis 2015; 15:62. 25888317 10.1186/s12879-015-0812-4 PMC4332438

[9] Angus DC van der Poll T Severe sepsis and septic shock. N Engl J Med 2013; 369:840–51. 23984731 10.1056/NEJMra1208623

[10] Cavaillon JM Adib-Conquy M Bench-to-bedside review: endotoxin tolerance as a model of leukocyte reprogramming in sepsis. Crit Care 2006; 10:233. 17044947 10.1186/cc5055 PMC1751079

[11] Hotchkiss RS Opal S Immunotherapy for sepsis—a new approach against an ancient foe. N Engl J Med 2010; 363:87–9. 20592301 10.1056/NEJMcibr1004371 PMC4136660

[12] Waitt CJ Peter K Banda N White SA Early deaths during tuberculosis treatment are associated with depressed innate responses, bacterial infection, and tuberculosis progression. J Infect Dis 2011; 204:358–62. 21742833 10.1093/infdis/jir265 PMC3132140

[13] Bisson GP Zetola N Collman RG Persistent high mortality in advanced HIV/TB despite appropriate antiretroviral and antitubercular therapy: an emerging challenge. Curr HIV/AIDS Rep 2015; 12:107–16. 25772785 10.1007/s11904-015-0256-x PMC5174096

[14] Ravimohan S Tamuhla N Steenhoff AP Immunological profiling of tuberculosis-associated immune reconstitution inflammatory syndrome and non-immune reconstitution inflammatory syndrome death in HIV-infected adults with pulmonary tuberculosis starting antiretroviral therapy: a prospective observational cohort study. Lancet Infect Dis 2015; 15:429–38. 25672566 10.1016/S1473-3099(15)70008-3 PMC4624391

[15] Western Cape Provincial AIDS Council, South Africa. Annual Progress Report 2014–2015. 2016(3).

[16] Benjamini Y Hochberg Y Controlling the false discovery rate: a practical and powerful approach to multiple testing. J Royal Stat Soc Series B. 1995; 57:289–300.

[17] Boulware DR Hullsiek KH Puronen CE; INSIGHT Study Group Higher levels of CRP, D-dimer, IL-6, and hyaluronic acid before initiation of antiretroviral therapy (ART) are associated with increased risk of AIDS or death. J Infect Dis 2011; 203:1637–46. 21592994 10.1093/infdis/jir134 PMC3096784

[18] Amancio RT Japiassu AM Gomes RN The innate immune response in HIV/AIDS septic shock patients: a comparative study. PLoS One 2013; 8:e68730. 23874739 10.1371/journal.pone.0068730 PMC3708901

[19] Bozza FA Salluh JI Japiassu AM Cytokine profiles as markers of disease severity in sepsis: a multiplex analysis. Crit Care 2007; 11:R49. 17448250 10.1186/cc5783 PMC2206478

[20] Aguilar-Ruiz SR Torres-Aguilar H González-Domínguez É Human CD16+ and CD16- monocyte subsets display unique effector properties in inflammatory conditions in vivo. J Leukoc Biol 2011; 90:1119–31. 21937707 10.1189/jlb.0111022

[21] Lugo-Villarino G Neyrolles O Dressed not to kill: CD16+ monocytes impair immune defence against tuberculosis. Eur J Immunol 2013; 43:327–30. 23322255 10.1002/eji.201243256

[22] Balboa L Romero MM Laborde E Impaired dendritic cell differentiation of CD16-positive monocytes in tuberculosis: role of p38 MAPK. Eur J Immunol 2013; 43:335–47. 23192690 10.1002/eji.201242557

[23] Monneret G Lepape A Voirin N Persisting low monocyte human leukocyte antigen-DR expression predicts mortality in septic shock. Intensive Care Med 2006; 32:1175–83. 16741700 10.1007/s00134-006-0204-8

[24] Lewis DK Peters RP Schijffelen MJ Clinical indicators of mycobacteraemia in adults admitted to hospital in Blantyre, Malawi. Int J Tuberc Lung Dis 2002; 6:1067–74. 12546114

[25] Wallis RS Hafner R Advancing host-directed therapy for tuberculosis. Nat Rev Immunol 2015; 15:255–63. 25765201 10.1038/nri3813

[26] Jarvis JN Meintjes G Rebe K Adjunctive interferon-γ immunotherapy for the treatment of HIV-associated cryptococcal meningitis: a randomized controlled trial. AIDS 2012; 26:1105–13. 22421244 10.1097/QAD.0b013e3283536a93 PMC3640254

[27] Delsing CE Gresnigt MS Leentjens J Interferon-gamma as adjunctive immunotherapy for invasive fungal infections: a case series. BMC Infect Dis 2014; 14:166. 24669841 10.1186/1471-2334-14-166 PMC3987054

[28] Pickkers PL The Effects of Interferon-gamma on Sepsis-induced Immunoparalysis https://clinicaltrialsgov/ct2/show/NCT01649921 Accessed 1 November 2015.

[29] Dawson R Condos R Tse D Immunomodulation with recombinant interferon-gamma1b in pulmonary tuberculosis. PLoS One 2009; 4:e6984. 19753300 10.1371/journal.pone.0006984 PMC2737621

[30] Bo L Wang F Zhu J Li J Deng X Granulocyte-colony stimulating factor (G-CSF) and granulocyte-macrophage colony stimulating factor (GM-CSF) for sepsis: a meta-analysis. Crit Care 2011; 15:R58. 21310070 10.1186/cc10031 PMC3221991

