# Supplementary figures and images for: Mortality in Severe Human Immunodeficiency Virus-Tuberculosis Associates With Innate Immune Activation and Dysfunction of Monocytes
Source: Clin Infect Dis. 2017 Mar 24;65(1):73–82. doi: 10.1093/cid/cix254 (PMC5849097; doi:10.1093/cid/cix254)

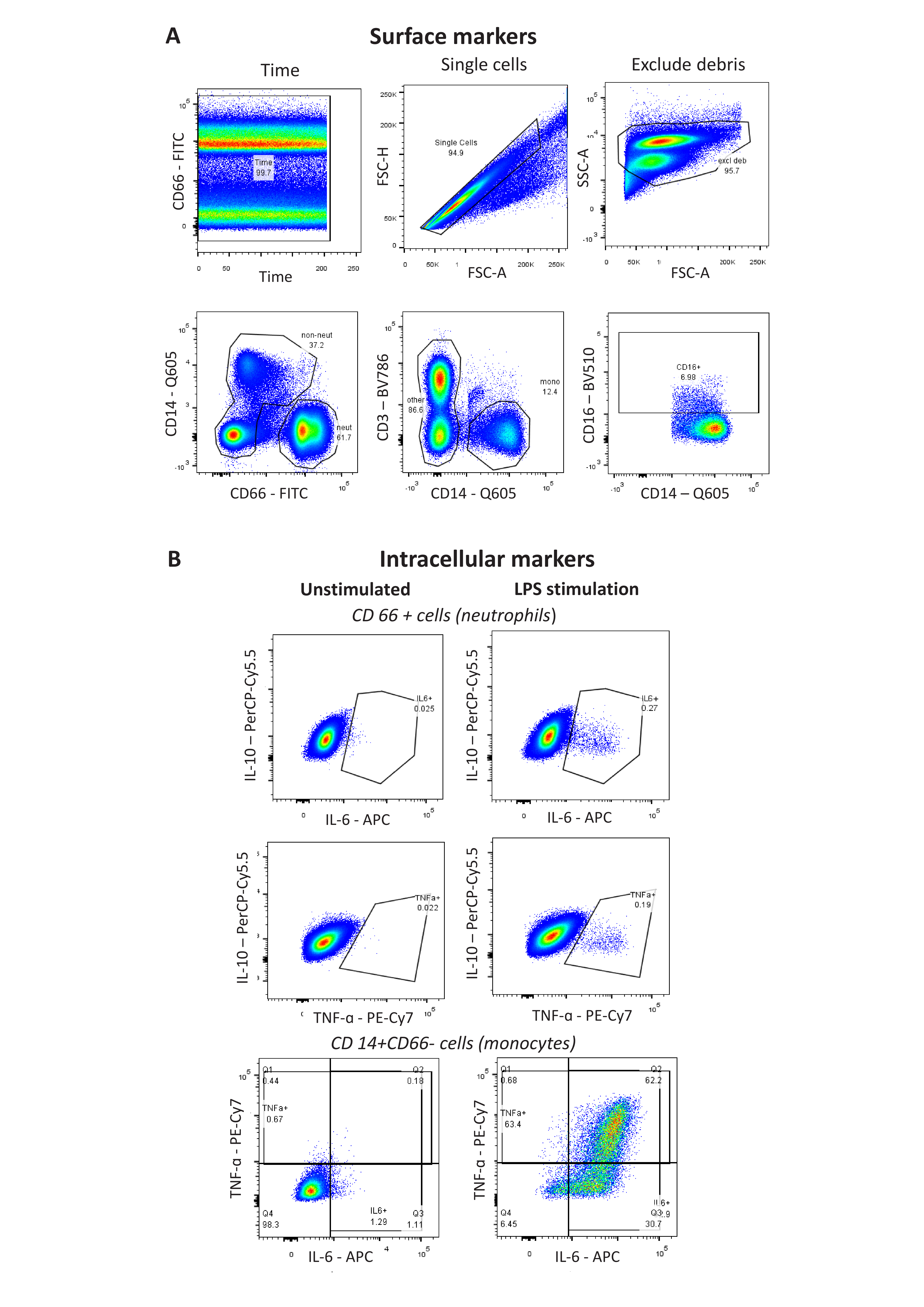

Supplement: Suppl_Fig_1 [file cix254_suppl_suppl_fig_1.png]

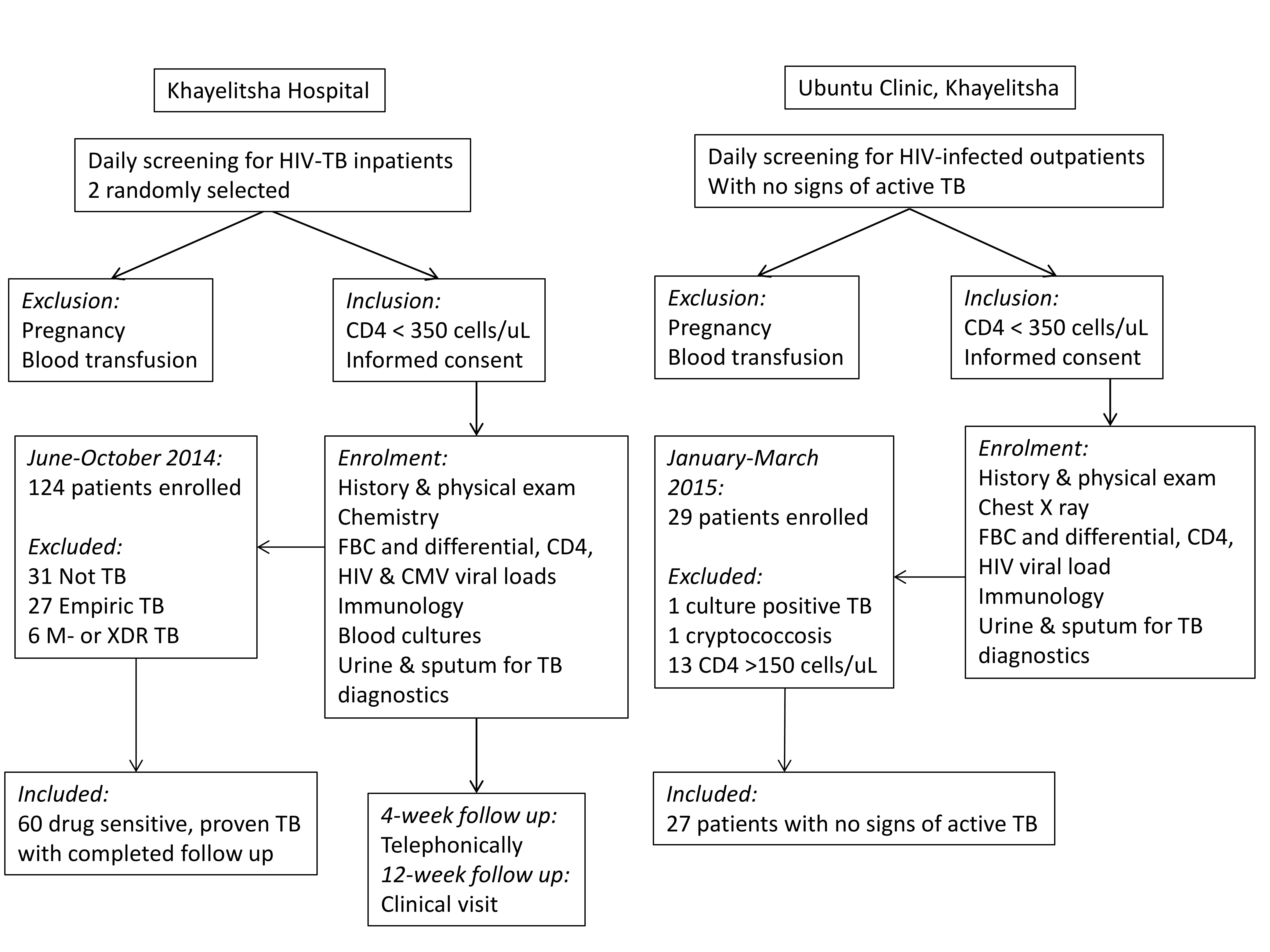

Supplement: Suppl_Fig_2 [file cix254_suppl_suppl_fig_2.png]

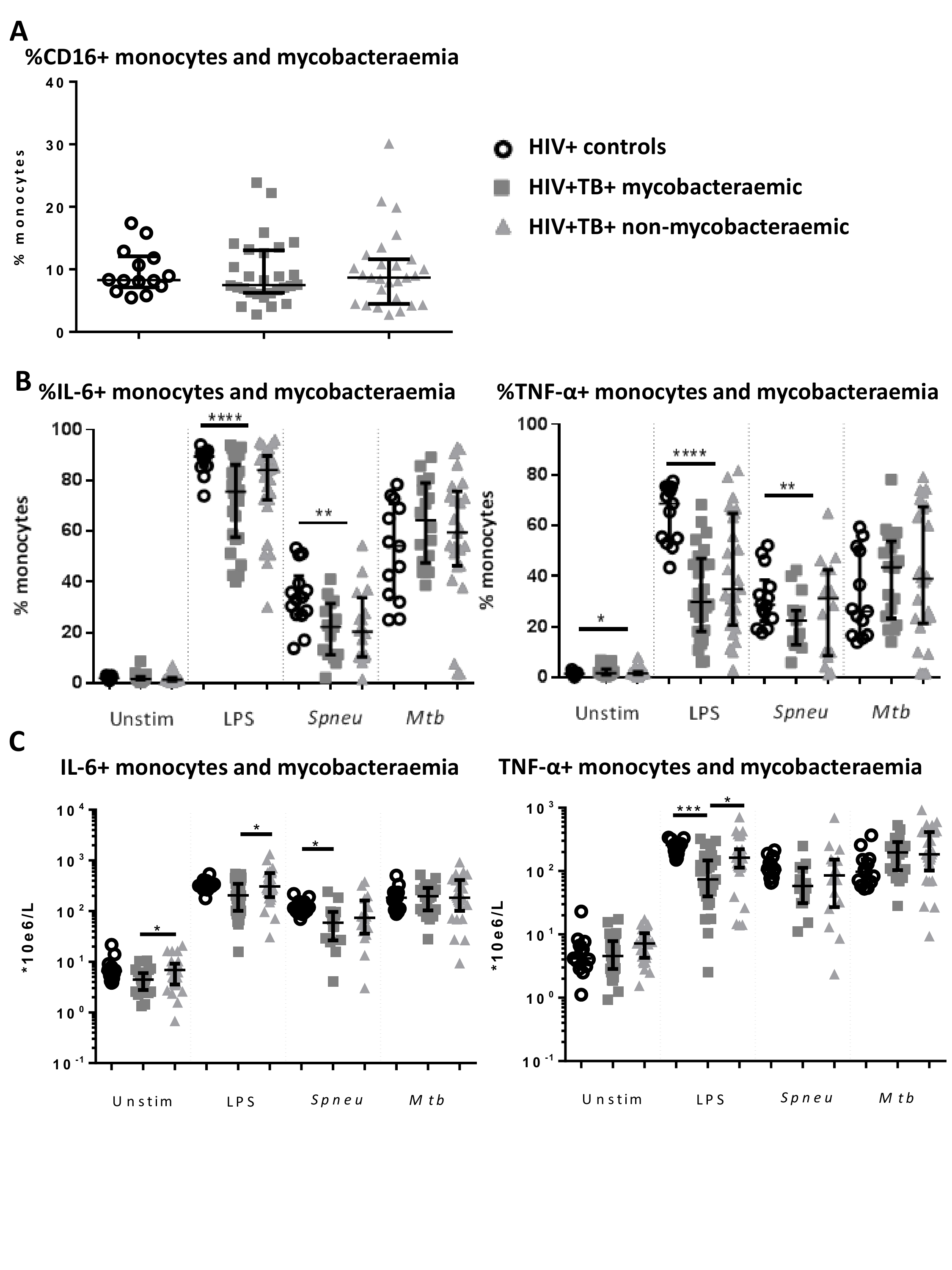

Supplement: Suppl_Figure_3 [file cix254_suppl_suppl_figure_3.png]
