# Supplementary material for: Mortality in Severe Human Immunodeficiency Virus-Tuberculosis Associates With Innate Immune Activation and Dysfunction of Monocytes
Source: Clin Infect Dis. 2017 Mar 24;65(1):73–82. doi: 10.1093/cid/cix254 (PMC5849097; doi:10.1093/cid/cix254)
Supplement: Supplementary_Table_1 [file cix254_suppl_supplementary_table_1.docx]

**Supplementary Table 1 Adapted sepsis criteria**

| **Sepsis** | **Documented infection + ≥ 1 of the following** |
| --- | --- |
| Fever or hypothermia | Tympanic temperature < 36°C or > 38°C |
| Tachycardia | Pulse rate > 90/minute |
| Tachypnea | Respiratory rate > 20/minute |
| Hypotension | Blood pressure < 90 mmHg |
| Altered mental state | Glasgow Coma Score < 15 |
| Hyperglycemica | Glucose > 6.7 mmol/L |
| Leukocytosis/leukopenia | White blood count > 12*10^9^/L or < 4*10^9^/L |
| Elevated plasma C-reactive protein | C-reactive protein > 100 mg/L |
| Elevated plasma procalcitonin | Procalcitonin > 2 µg/L |
|  |  |
| **Severe sepsis** | **Sepsis + organ dysfunction (≥ 1 of the following)** |
| Renal failure | Creatinin > 100 mmol/L |
| Hyperbilirubinemia | Bilirubin > 7 mmol/L |
| Thrombocytopenia | Platelets < 100*10^9^/L |
|  |  |
| **Septic shock** | **Sepsis + hyperlactatamia** |
| Hyperlactatemia | Lactate > 2.2 mmol/L |

Table showing case criteria for sepsis, severe sepsis and septic shock, adapted from previously published criteria[3]. Criteria were adapted to make them feasible in this sub-Saharan emergency centre setting. Criteria for organ dysfunction were simplified (increased creatinine, hyperbilirubinaemia and thrombocytopaenia), as no information was available on urine output, arterial hypoxaemia or coagulation abnormalities. Reliable information on fluid resuscitation and response to intravenous fluid therapy was not available. Therefore hyperlactataemia was used as a proxy for septic shock.
