# Supplementary material for: Mortality in Severe Human Immunodeficiency Virus-Tuberculosis Associates With Innate Immune Activation and Dysfunction of Monocytes
Source: Clin Infect Dis. 2017 Mar 24;65(1):73–82. doi: 10.1093/cid/cix254 (PMC5849097; doi:10.1093/cid/cix254)
Supplement: Supplementary_Table_2 [file cix254_suppl_supplementary_table_2.docx]

**Supplementary Table 2 Overview of the reagents used**

| **Reagent** | **Clones** | **Company** |
| --- | --- | --- |
| *Flow cytometry* | | |
| CD3-BV786 | Mouse IgG1, kappa | *BD Horizon, Belgium* |
| CD19-BV421 | Mouse IgG1, kappa | *BD Horizon, Belgium* |
| CD14-Q605 | Mouse IgG2a, kappa | *Invitrogen, California, USA* |
| CD16-BV510 | Mouse IgG1, kappa | *BD Horizon, Belgium* |
| CD56-PE-CF594 | Mouse IgG1, kappa | *BD Horizon, Belgium* |
| CD66a/c/e-FITC | Mouse IgG2a, kappa | *BD Horizon, Belgium* |
| TNFɑ*-PE-Cy7 | Mouse IgG1, kappa | *BD Horizon, Belgium* |
| IFN^†^-ɣ-Alexa Fluor 700 | Mouse IgG1, kappa | *BD Horizon, Belgium* |
| IL^‡^-6-APC | Rat IgG1, kappa | *Biolegend, California, USA* |
| IL^‡^-10-PerCP-Cy5.5 | Rat IgG2a, kappa | *Biolegend, California, USA* |
| IL^‡^-17A-PE | Mouse IgG1, kappa | *BD Horizon, Belgium* |
| *Luminex* | | |
| CSF^§^-2 | | *Merck Millipore, Massachusetts, USA* |
| CSF^§^-3 | | *Merck Millipore, Massachusetts, USA* |
| IFN^†^-ɑ2 | | *Merck Millipore, Massachusetts, USA* |
| IFN^†^-ɣ | | *Merck Millipore, Massachusetts, USA* |
| IL^‡^-10 | | *Merck Millipore, Massachusetts, USA* |
| IL^‡^-12p40 | | *Merck Millipore, Massachusetts, USA* |
| IL^‡^-1RA | | *Merck Millipore, Massachusetts, USA* |
| IL^‡^-1β | | *Merck Millipore, Massachusetts, USA* |
| IL^‡^-6 | | *Merck Millipore, Massachusetts, USA* |
| IL^‡^-7 | | *Merck Millipore, Massachusetts, USA* |
| IL^‡^-8 | | *Merck Millipore, Massachusetts, USA* |
| TNFɑ* | | *Merck Millipore, Massachusetts, USA* |

Overview of the reagents used for flow cytometry and Luminex assays.

*tumor necrosis factor-α (TNF-α); ^†^interferon (IFN); ^‡^ interleukin (IL); ^§^colony stimulating factor (CSF).
