# Supplementary material for: Mortality in Severe Human Immunodeficiency Virus-Tuberculosis Associates With Innate Immune Activation and Dysfunction of Monocytes
Source: Clin Infect Dis. 2017 Mar 24;65(1):73–82. doi: 10.1093/cid/cix254 (PMC5849097; doi:10.1093/cid/cix254)
Supplement: Supplementary_Table_3 [file cix254_suppl_supplementary_table_3.docx]

**Supplementary Table 3 Causes of death and contributing factors**

| **Age/gender** | **Time to from enrolment to TB treatment (days)** | **Time to death (days)** | **Admission episode at death** | **Cause of death** | **Contributing factors** |
| --- | --- | --- | --- | --- | --- |
| 62/F | 2 | 12 | Initial admission | Hypovolemic shock due to upper gastro-intestinal bleed | Abdominal TB * |
| 64/F | 8 | 13 | Initial admission | Bacterial sepsis; likely urogenital origin | Urine cultured ESBL^†^ *Klebsiella*  Leukocytosis and renal failure |
| 35/F | 1 | 20 | Readmission | Disseminated TB* | Dead on arrival to hospital |
| 36/F | 0 | 7 | Initial admission | Disseminated TB* | Renal failure and hyperkalaemia  Respiratory failure |
| 50/M | 0 | 0 | Initial admission | Disseminated TB* | Died after 1^st^ dose of TB* treatment |
| 57/F | 0 | 83 | Readmission | Advanced HIV and TB* | Diarrhoea |
| 58/F | 2 | 10 | Initial admission | Unproven bacterial sepsis; likely respiratory origin | Deterioration after admission  New infiltrate on chest X-ray |
| 35/M | 0 | 7 | Initial admission | Disseminated TB* | No improvement on TB* treatment |
| 43/F | 1 | 2 | Initial admission | Disseminated TB* | Died after 1^st^ dose of TB* treatment |
| 43/M | 0 | 35 | Readmission | Disseminated TB* | Fluctuant peri-umbilical mass, aetiology uncertain |
| 46/F | 3 | 6 | Initial admission | Disseminated TB* | Died after 2 doses of TB* treatment |
| 22/F | 1 | 34 | Readmission | Neurological TB* IRIS^‡^ | Disseminated TB*, improved on TB* treatment  Neurologic deterioration after ART^§^ was started |
| 45/M | 0 | 3 | Initial admission | Disseminated TB* | Renal failure secondary to TB* or tenofovir  Tenofovir was stopped, no improvement |
| 35/M | 1 | 11 | Readmission | Unproven bacterial sepsis; likely respiratory origin  Unconfirmed PE^II^ | Respiratory failure  New infiltrate on chest X ray  DVT** and clinical/ultrasonographic signs of PE^II^ for which thrombolysis was given |
| 46/F | 2 | 75 | Readmission | Disseminated TB*  Multi-organ failure due to bacterial sepsis, IRIS^‡^ or drug toxicity | Confusion, hypovolemic shock, progressive renal failure, liver failure, disseminated intravascular coagulation  Few weeks after starting ART^§^ |
| 43/M | 0 | 20 | Initial admission | Renal failure due to drug toxicity and disseminated TB* | Recently started tenofovir |

Table 2 shows the most likely causes and contributing factors to death.

*Tuberculosis (TB); ^†^Extended Spectrum Beta Lactamase (ESBL); ^‡^immune reconstitution inflammatory syndrome (IRIS); ^§^antiretroviral therapy (ART); ^II^pulmonary embolism (PE); **deep venous thrombosis (DVT)
