# Supplementary material for: Mortality in Severe Human Immunodeficiency Virus-Tuberculosis Associates With Innate Immune Activation and Dysfunction of Monocytes
Source: Clin Infect Dis. 2017 Mar 24;65(1):73–82. doi: 10.1093/cid/cix254 (PMC5849097; doi:10.1093/cid/cix254)
Supplement: Supplementary_Table_4 [file cix254_suppl_supplementary_table_4.docx]

**Supplementary Table 4 Cytokine concentrations in culture supernatants of mycobacteremic patients versus non-mycobacteremic patients**

|  | **Non-mycbacteremic HIV+TB+** | | **Mycobacteremic HIV+ TB+** | |  |
| --- | --- | --- | --- | --- | --- |
| **Unstimulated** | | | | | |
| **Cytokine** | **Median** | **IQR*** | **Median** | **IQR*** | **q-value** |
| CSF^†^-3 | 73.3 | 50.6-155.7 | 110.0 | 52.4-183 | 0.89 |
| CSF^†^-2 | 12.3 | 10.9-13.7 | 11.6 | 10.4-14.5 | 0.77 |
| IFN^‡^-A2 | 12.2 | 10.2-16.6 | 15.9 | 11.1-19.3 | 0.77 |
| IFN^‡^-ɣ | 74.5 | 36.7-262.1 | 141.3 | 44.9-249.9 | 0.77 |
| IL^§^-10 | 20.7 | 13.3-31.8 | 25.2 | 17.6-27.8 | 0.77 |
| IL^§^-12p40 | 19.9 | 15.7-22.6 | 19.9 | 16.3-26.4 | 0.88 |
| IL^§^-1RA | 237.5 | 107.9-490.2 | 292.7 | 109.5-496.1 | 0.93 |
| IL^§^-1β | 3.86 | 2.7-5.0 | 2.81 | 2.39-4.41 | 0.77 |
| IL^§^-6 | 47.9 | 23.7-110.3 | 51.0 | 23.5-78.1 | 0.99 |
| IL^§^-8 | 626.1 | 302.8-2485 | 604.8 | 188.2-1124 | 0.77 |
| TNF-α^II^ | 119.9 | 66.0-159.3 | 89.5 | 55.5-152.5 | 0.77 |
| **LPS** | | | | | |
| CSF^†^-3 | 439.9 | 286.6-806.4 | 303.3 | 127.2-564.1 | 0.26 |
| CSF^†^-2 | 12.7 | 11.0-14.1 | 13.8 | 11.5-16.1 | 0.77 |
| IFN^‡^-A2 | 13.8 | 11.3-17.6 | 17.1 | 12.3-21.9 | 0.77 |
| IFN^‡^-ɣ | 79.2 | 46.5-198.7 | 127.1 | 52.4-221.7 | 0.77 |
| IL^§^-10 | 318.9 | 177.2-630.2 | 118.8 | 54.0-283.7 | 0.22 |
| IL^§^-12p40 | 37.7 | 28.1-117.8 | 40.8 | 23.7-77.4 | 0.77 |
| IL^§^-1RA | 2542 | 1159-5411 | 1819 | 652-4163 | 0.77 |
| IL^§^-1β | 567.3 | 116.7-1898 | 234.2 | 36.8-443.8 | 0.22 |
| IL^§^-6 | 8600 | 3215-10128 | 4832 | 1528-8822 | 0.26 |
| IL^§^-8 | 6446 | 2303-9878 | 2550 | 1252-9037 | 0.44 |
| TNF-α^II^ | 3111 | 1154-6917 | 1299 | 665.7-3043 | 0.26 |
| ***S. pneumoniae*** | | | | | |
| CSF^†^-3 | 154.8 | 102.1-227.2 | 129.3 | 85.3-243.7 | 0.77 |
| CSF^†^-2 | 12.5 | 11.3-14.5 | 12.7 | 11.4-14.8 | 0.99 |
| IFN^‡^-A2 | 13.8 | 11.4-18.2 | 13.5 | 12.2-22.0 | 0.99 |
| IFN^‡^-ɣ | 73.2 | 43.0-196.6 | 128.8 | 56.2-219.4 | 0.77 |
| IL^§^-10 | 42.8 | 21.8-88.0 | 34.9 | 28.2-67.5 | 0.93 |
| IL^§^-12p40 | 23.4 | 17.4-38.0 | 28.6 | 22.3-33.5 | 0.77 |
| IL^§^-1RA | 903.3 | 371.9-1789 | 797.2 | 582.6-1935 | 0.77 |
| IL^§^-1β | 134.4 | 39.4-341 | 67.0 | 11.3-202.5 | 0.77 |
| IL^§^-6 | 1647 | 706.1-4496 | 1145 | 245.9-3631 | 0.77 |
| IL^§^-8 | 9301 | 3935-11352 | 7235 | 1394-10727 | 0.77 |
| TNF-α^II^ | 1963 | 483.7-4974 | 894.3 | 471.8-2785 | 0.77 |
| ***M. tuberculosis* (H37rv)** | | | | | |
| CSF^†^-3 | 378 | 233.8-942.6 | 420.3 | 153.4-861.8 | 0.93 |
| CSF^†^-2 | 23.5 | 17.6-30.9 | 18.4 | 13.3-37.1 | 0.77 |
| IFN^‡^-A2 | 13.8 | 11.5-18.2 | 15.8 | 12.3-21.9 | 0.77 |
| IFN^‡^-ɣ | 101.8 | 38.4-295.5 | 150.6 | 66.3-286.8 | 0.77 |
| IL^§^-10 | 92.7 | 45.5-259.5 | 93.9 | 48.0-307.4 | 0.93 |
| IL^§^-12p40 | 23.7 | 16.3-37.1 | 27.2 | 19.9-37.3 | 0.77 |
| IL^§^-1RA | 877.3 | 282-1337 | 832.8 | 441.5-1634 | 0.77 |
| IL^§^-1β | 407.2 | 50.7-1315 | 449.4 | 37.7-1076 | 0.77 |
| IL^§^-6 | 7893 | 1524-11140 | 3684 | 1588-9823 | 0.82 |
| IL^§^-8 | 9303 | 5121-11174 | 9291 | 2689-11642 | 0.93 |
| TNF-α^II^ | 2504 | 747.2-8021 | 2187 | 757.6-6366 | 0.77 |

Median and interquartile ranges of cytokines concentrations measured in culture supernatants of HIV-TB patients with mycobacteremia and those with negative mycobacterial blood cultures, respectively. Values are in picogram per millilitre. Mann-Whitney U tests were used for non-parametric data, Students’ T-tests for parametric data.

* Interquartile range (IQR); ^†^colony stimulating factor (CSF); ^‡^interferon (IFN); ^§^interleukin (IL); ^II^tumor necrosis factor-α (TNF-α); **lipopolysaccharide (LPS).
