# Supplementary material for: Mortality in Severe Human Immunodeficiency Virus-Tuberculosis Associates With Innate Immune Activation and Dysfunction of Monocytes
Source: Clin Infect Dis. 2017 Mar 24;65(1):73–82. doi: 10.1093/cid/cix254 (PMC5849097; doi:10.1093/cid/cix254)
Supplement: Paper_mycobacteremia_OnlineDataSuppl_CID_acc_changes [file cix254_suppl_paper_mycobacteremia_onlinedatasuppl_cid_acc_changes.docx]

**Mortality in Severe HIV-TB Associates with Innate Immune Activation and Dysfunction of Monocytes**

*Running head: Innate Immunity and Mortality in Severe HIV-TB*

Saskia Janssen, Charlotte Schutz, Amy Ward, Elisa Nemes, Katalin A. Wilkinson, James Scriven, Mischa A. Huson, Nanne Aben, Gary Maartens, Rosie Burton, Robert J. Wilkinson, Martin P. Grobusch, Tom Van der Poll, Graeme Meintjes

**Online Data Supplement**

**SUPPLEMENTARY METHODS**

**Procedures**

For HIV-TB patients, sputum and urine samples were collected and processed as previously described [1]. The Xpert® MTB/RIF assay was performed on concentrated urine samples (Cepheid, Sunnyvale, CA, USA). Sputum (spontaneous or induced), when produced, was sent for Xpert® MTB/RIF and TB culture. MycoFlytic blood culture (BC) bottles were inoculated with 5 mL whole blood and cultured until positive, or for 42 days if negative; a positive result was identified as *M. tuberculosis* (*Mtb*) using GenoType® MTBDRplus (Hain Lifescience, Nehren, Germany); rifampicin and isoniazid sensitivity testing was done on all positive cultures. Full blood counts and differentials (Roche XN-10 Sysmex, Norderstedt, Germany), chemistry (C-reactive protein (CRP), glucose, lactate, procalcitonin and albumin; Roche Cobas 6000, Mannheim, Germany), HIV viral loads (Abbott M2000 SP/RT, Wiesbaden, Germany) and CD4 counts (Beckman Coulter FC 500 Analysis Cellmek Preparation, Milan, Italy) were performed at the National Health Laboratory Service (NHLS) laboratory. Blood for immunology assays (described below) was taken at enrolment. Patients were followed up telephonically at 4 weeks and clinically reviewed at 12 weeks. If no contact could be established, regional laboratory and pharmacy information systems were used to ascertain vital status at 12 weeks. Patients were managed by the hospital doctors and not study staff.

For HIV-infected control outpatients without active TB (controls) a symptom screen [2], physical examination and chest-X ray were performed along with CD4 count, HIV-viral load, full blood count and differential. Sputum (spontaneous or induced), was sent for Xpert® MTB/RIF and TB culture, and urine Xpert® MTB/RIF was performed in all. Controls were excluded if symptom screen or any TB diagnostic tests was positive.

**Ethics**

Ethics approval was obtained from the University of Cape Town Faculty of Health Sciences Human Research Ethics Committee (UCT FHS HREC reference number 057/2013 and 568/2014). Written informed consent was sought from all patients. Patients who were initially too ill to provide consent were enrolled and monitored daily. Patients were invited to provide informed consent or withdraw from the study once they regained capacity to consent. If a patient died prior to obtaining consent, we obtained specific permission from the UCT FHS HREC to include these patients’ data.

**Outcomes**

The primary outcome was all-cause mortality at 12-weeks. We aimed to determine immunologic changes associated with 12-week mortality. In a secondary analysis, we assessed the association of TB mycobacteremia, defined as at least 1 MycoFlytic blood culture growing *Mtb,* with immunologic profile and outcome.

**Data sources and measurement**

**Clinical data**

Clinical data were captured from patient files, history and physical exam. Laboratory results were captured from the online NHLS database. The cause of death was assessed using clinical notes, as well as personal communication with the patient’s physician and/or relatives. Clinical notes of all deaths were discussed by four of the authors (SJ, CS, AW, G Meintjes) and consensus was reached regarding the most probable cause of death. We used definitions for sepsis, severe sepsis, and septic shock adapted from previously published criteria (Supplementary Table 1)[3].

**Ex vivo whole blood stimulation experiments**

Heparinized whole blood was obtained at enrolment and stimulated within 2 hours using a mobile incubator (CryoLogic, Biotherm, Blackburn, Victoria, Australia), for six hours in total. Six different stimulation conditions were set up in duplicate, whenever logistically possible, to assess cytokine production intracellularly and in the supernatant (Supplementary Table 2). Anti-bacterial responses were tested using *E. coli* derived lipopolysaccharide (LPS) (1 µg/mL, Invivogen, San Diego, CA, USA) and heat-killed *Streptococcus pneumoniae*, strain ATCC 6303 (1.8*10^8^ CFU/mL, Davies Diagnostics, Johannesburg, South Africa)*.* Anti-mycobacterial responses were tested using heat-killed *Mtb* strain H37Rv (7.1*10e5 CFU/mL, in-house). The effect of IFN-ɣ (1 ng/mL, RnD Systems, Minneapolis, MN, USA) was assessed in a co-stimulation assay with LPS. Phosphate buffered saline (PBS, un-stimulated) and human recombinant IFN-ɣ without LPS were used as appropriate controls. Concentrations for all conditions were tested in dose-response experiments, except for recombinant IFN-ɣ (the concentration used was based on serum levels achieved *in vivo* in a clinical trial [4]). For measurement of intracellular cytokines, brefeldin A (16 µg/mL, Sigma-Aldrich Corp., St. Louis, MO, USA) was added to the stimulated blood after 2 hours; white blood cells were harvested after 6 hours as described before [5]. Cells were stored in liquid nitrogen until further analysis by flow cytometry. For measurement of secreted cytokines, supernatants were collected at 6 hours of stimulation and stored at -80°C.

**Measurement of intracellular cytokines**

Cells were thawed and stained with surface markers (CD3, CD14, CD16, CD19, CD56, CD66; Table E2) for 30 minutes at 4°C. Thereafter, cells were permeabilised with PermWash (BD Horizon, Erembodegem, Belgium) and stained with antibodies for intracellular cytokines (IL-6 and tumour necrosis factor-ɑ (TNF-ɑ); Supplementary Table 2) for 30 minutes at 4°C. Samples were fixed with 4% paraformaldehyde prior to acquisition on a BD LSR Fortessa Flow Cytometer configured daily with CS&T beads (BD Biosciences, Erembodegem, Belgium), using a forward scatter threshold of 30,000. Where possible, 800,000 events were recorded. Samples with less than 250,000 events were excluded from analyses. Positive and negatively labelled capture beads (BD Horizon, Erembodegem, Belgium) were used to calculate daily compensation. Fluorescence minus one experiments were conducted to assess the panel and adjust gating considering cell auto-fluorescence, and isotype controls for surface markers were used to control for non-specific binding.

Data were analysed in FlowJo version 10 (Ashland, OR, USA). Gating strategies are illustrated in Supplementary Figure 1. After excluding doublets and debris, neutrophils were gated on CD66a/c/e. On the remaining cells, CD14 was used to identify monocytes. CD14+ cells were divided into CD16+ and CD16- subsets in unstimulated samples. We did intracellular staining for IL-6 and TNF-ɑ in monocytes and neutrophils. To determine cytokine production, gates were drawn on unstimulated samples, and the unstimulated value was subtracted from the stimulated value to determine the absolute change. Absolute counts of cytokine producing cell subsets were derived by multiplying the percentage obtained with flow cytometry with the cell count of the respective population obtained from the NHLS laboratory differential count (Roche XN-10 Sysmex, Norderstedt, Germany).

**Measurement of cytokines in culture supernatants**

The concentrations of 12 cytokines (colony stimulating factor 2 (CSF-2; also known as granulocyte monocyte colony stimulating factor), colony stimulating factor 3 (CSF-3; also known as granulocyte colony stimulating factor), interferon- α2 (IFN-α2), IFN-ɣ, IL-1-receptor antagonist (IL-1RA), IL-1β, IL-6, IL-7, IL-8, IL-10, IL-12p40, TNF-ɑ; Supplementary Table 2) were measured in culture supernatants (heparin anticoagulated plasma) using a commercial Luminex multiplex assay (Merck Millipore, Boston, MA, USA). Samples were acquired undiluted on a Bio-Plex 200 system (Bio-Rad, Waltham, MA, USA). IL-7 was consistently undetectable and therefore not analysed. The quality control (provided with the assay) was passed for all measured cytokines, except for IFNα2, where the quality controls consistently fell slightly below the range provided with the assay. Standard curves were fitted using GraphPad Prism, version 6 (San Diego, CA, USA).

Bias in laboratory assays was minimised by randomly including samples from patients from all groups (HIV-associated TB with mycobacteremia, HIV-associated TB without mycobacteremia, surviving and deceased patients, and HIV-infected control patients) on each acquisition day (flow cytometry) or plate (Luminex).

**REFERENCES**

1. Lawn SD, Kerkhoff AD, Burton R, Schutz C, van Wyk G, Vogt M, Pahlana P, Nicol MP, Meintjes G. Rapid microbiological screening for tuberculosis in HIV-positive patients on the first day of acute hospital admission by systematic testing of urine samples using Xpert MTB/RIF: a prospective cohort in South Africa. BMC Med. **2015**;13:192.

2. Getahun H, Kittikraisak W, Heilig CM, Corbett EL, Ayles H, Cain KP, Grant AD, Churchyard GJ, Kimerling M, Shah S, Lawn SD, Wood R, Maartens G, Granich R, Date AA, Varma JK. Development of a standardized screening rule for tuberculosis in people living with HIV in resource-constrained settings: individual participant data meta-analysis of observational studies. PLoS Med. **2011**;8(1):e1000391.

3. Angus DC, van der Poll T. Severe sepsis and septic shock. N Engl J Med. **2013**;369(9):840-51.

4. Pickkers PL. The Effects of Interferon-gamma on Sepsis-induced Immunoparalysis. https://clinicaltrialsgov/ct2/show/NCT01649921 Accessed 01-11-2015.

5. Shey MS, Hughes EJ, de Kock M, Barnard C, Stone L, Kollmann TR, Hanekom WA, Scriba TJ. Optimization of a whole blood intracellular cytokine assay for measuring innate cell responses to mycobacteria. J Immunol Meth. **2012**;376(1-2):79-88.

**SUPPLEMENTARY TABLE LEGENDS**

**Supplementary Table 1 Adapted sepsis criteria**

Table showing case criteria for sepsis, severe sepsis and septic shock, adapted from previously published criteria[3]. Criteria were adapted to make them feasible in this sub-Saharan emergency centre setting. Criteria for organ dysfunction were simplified (increased creatinine, hyperbilirubinaemia and thrombocytopaenia), as no information was available on urine output, arterial hypoxaemia or coagulation abnormalities. Reliable information on fluid resuscitation and response to intravenous fluid therapy was not available. Therefore hyperlactataemia was used as a proxy for septic shock.

**Supplementary Table 2 Overview of the reagents used**

Overview of the reagents used for flow cytometry and Luminex assays.

*tumor necrosis factor-α (TNF-α); ^†^interferon (IFN); ^‡^ interleukin (IL); ^§^colony stimulating factor (CSF).

**Supplementary Table 3 Causes of death and contributing factors**

Supplementary Table 3 shows the most likely causes and contributing factors to death.

*Tuberculosis (TB); ^†^Extended Spectrum Beta Lactamase (ESBL); ^‡^immune reconstitution inflammatory syndrome (IRIS); ^§^antiretroviral therapy (ART); ^II^pulmonary embolism (PE); **deep venous thrombosis (DVT)

**Supplementary Table 4 Cytokine concentrations in culture supernatants of mycobacteremic patients versus non-mycobacteremic patients**

Median and interquartile ranges of cytokines concentrations measured in culture supernatants of HIV-TB patients with mycobacteremia and those with negative mycobacterial blood cultures, respectively. Values are in picogram per millilitre. Mann-Whitney U tests were used for non-parametric data, Students’ T-tests for parametric data.

* Interquartile range (IQR); ^†^colony stimulating factor (CSF); ^‡^interferon (IFN); ^§^interleukin (IL); ^II^tumor necrosis factor-α (TNF-α); **lipopolysaccharide (LPS).

**SUPPLEMENTARY FIGURE LEGENDS**

**Supplementary Figure 1. Gating strategy**

The gating strategy used for flow cytometry is shown. (A) After excluding doublets and debris, neutrophils were gated on CD66a/c/e. On the remaining cells, CD14 was used to identify monocytes. (B) We included IL-6 and TNF-ɑ production in monocytes and neutrophils in our analyses.

**Supplementary Figure 2. Study population**

Flow diagram showing an overview of methodology, patient recruitment and inclusion in this study.

Non-pregnant HIV-infected patients with CD4 counts < 350 cells/µL, diagnosed with TB or with a high clinical suspicion of TB on admission to Khayelitsha Hospital were recruited between June and November 2014. Patients who received a blood transfusion were excluded. Only those patients with microbiologically proven rifampicin-susceptible TB were included in the analyses. On weekdays, a list was compiled of all patients fulfilling inclusion criteria in the emergency and medical wards. Through a random selection procedure, up to two patients were enrolled from the list each day. HIV-infected outpatients with CD4 counts < 350 cells/ µL without active TB were recruited at Ubuntu clinic in Khayelitsha as controls.

**Supplementary Figure 3. Monocyte responses and TB mycobacteremia**

Median values and interquartile ranges are shown for the percentage of CD16+ monocytes in unstimulated samples (A), and percentages (B) and absolute counts (C) of IL-6+ and TNF-ɑ+ monocytes in response to respective stimulants. Absolute counts were derived by multiplying the percentage of positive cells by the monocyte count obtained from the NHLS clinical laboratory. HIV-infected controls (black circles), HIV-TB patients with mycobacteremia (dark grey squares) and non-mycobacteremic HIV-TB patients (light grey triangles) are shown. Kruskall-Wallis and Mann-Whitney-U tests were used for comparisons between groups; HIV-infected controls were compared to HIV-TB patients, and mycobacteremic HIV-TB patients were compared to non-mycobacteremic HIV-TB patients. *p-value < 0.05, **p-value < 0.01, *** p-value < 0.001, ****p-value < 0.0001.
